# Multilayer-Coated Tablet of Clopidogrel and Rosuvastatin: Preparation and In Vitro/In vivo Characterization

**DOI:** 10.3390/pharmaceutics11070313

**Published:** 2019-07-04

**Authors:** Ki-Soo Seo, Hyo-Kyung Han

**Affiliations:** 1 College of Pharmacy, Dongguk University-Seoul, Dongguk-ro-32, Ilsan-Donggu, Goyang 10326, Korea; 2 Research Institute, Dong Wha Pharm., Tapsil-ro-35, Giheung-gu, Yongin 17084, Korea

**Keywords:** acid instability, multilayered tablet, surface coating, combination, dissolution, pharmacokinetics

## Abstract

The acid lability of rosuvastatin hinders the preparation of mixed combination formulations of rosuvastatin with acidic drugs such as clopidogrel. Therefore, the purpose of this study was to develop a multilayer-coated tablet that avoids physicochemical interactions between rosuvastatin and clopidogrel. Among the tested hydrophobic materials, glyceryl behenate was most effective at inhibiting the production of lactone, the acid degradation product of rosuvastatin. Therefore, the multilayer-coated tablet included a hydrophobic separation layer consisting of glyceryl behenate between the clopidogrel core tablet and the rosuvastatin coating layer. In order to prevent delayed dissolution by the stable hydrophobic separation layer, crospovidone was added into the clopidogrel core tablet as an effective disintegrant. Copovidone was also added to the coating layer of rosuvastatin, achieving a dissolution profile comparable to that of the reference drug, Crestor^®^. The resulting multilayer-coated tablet exhibited similar pharmacokinetic profiles to those of reference drugs (Plavix^®^ and Crestor^®^) in beagle dogs, and there was no statistically significant difference in the maximum plasma concentration (*C*_max_), the time to reach the maximum plasma concentration (*T*_max_), or the area under the plasma-concentration time curve (*AUC*) between the test and reference formulations. The storage stability tests showed that the amounts of acid degradation products and total impurities were comparable to that of the reference drug. In conclusion, the present study successfully developed a stable multilayer-coated tablet containing both clopidogrel and rosuvastatin that may improve the patient compliance in combination therapy for cardiovascular diseases.

## 1. Introduction

**Figure 1:**
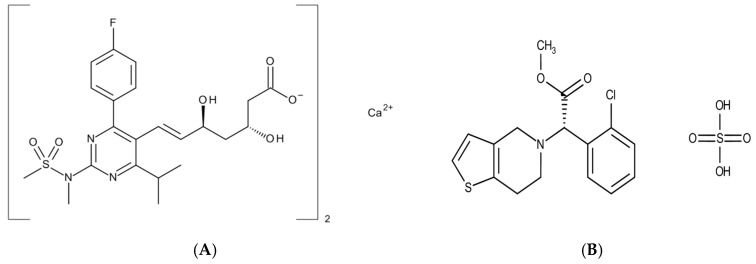
Chemical structures of rosuvastatin calcium (**A**) and clopidogrel bisulfate (**B**).

3-hydroxy-3-methyl-glutaryl-coenzyme A (HMG-CoA) reductase inhibitors are the most commonly used antidyslipidemic agents, lowering both total cholesterol and low-density lipoprotein (LDL) cholesterol levels while increasing high-density lipoprotein (HDL) cholesterol levels [1,2]. Cholesterol biosynthesis is decreased by inhibiting the activity of HMG-CoA reductase, the first rate-determining enzyme, which catalyzes the conversion of HMG-CoA into mevalonate, a cholesterol precursor [3]. This decrease in liver cholesterol synthesis up-regulates LDL receptors and lowers plasma LDL levels via the increased clearance of LDL from plasma [4]. One of the most commonly prescribed HMG-CoA reductase inhibitors is rosuvastatin calcium ([(3R,5S,6E)-7-{4-(4-fluorophenyl)-6-isopropyl-2-[methyl(methylsulfonyl)amino]-5-pyrimidinyl}-3,5-dihydroxy-6-heptenoate] calcium salt (2:1)), which is shown in Figure 1A [5,6]. Since rosuvastatin is easily degraded in acidic conditions, formulating rosuvastatin with other acidic active ingredients or acid salt substitutes causes instability issues. A representative acid hydrolysis product is (3R, 5S) lactone, which is produced by the oxidation of a hydroxyl group adjacent to a carbon–carbon double bond to a ketone functional group in an acidic environment [7,8].

**Figure 2:**
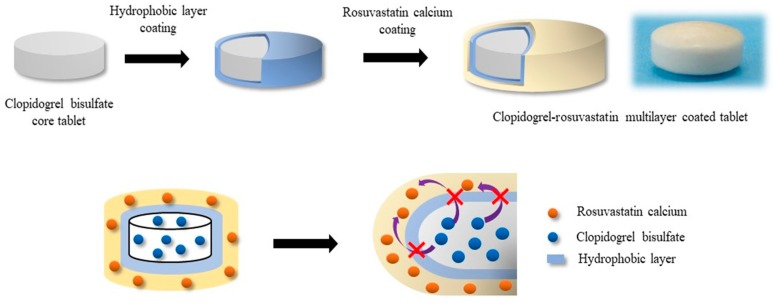
Structural illustration of the multilayer-coated tablet containing clopidogrel and rosuvastatin.

Thrombosis is the main complication of atherosclerosis, which is caused by platelet activation and aggregation from the disruption of an atherosclerotic plaque [9]. As an antiplatelet drug, clopidogrel (methyl (2S)-2-(2-chlorophenyl)-2-(6, 7-dihydro-4H-thieno[3, 2-c]pyridin-5-yl) acetate) (Figure 1B) inhibits the binding of adenosine diphosphate (ADP) to its platelet P2Y12 receptor. This, in turn, inhibits ADP-mediated activation of the glycoprotein GPIIb/IIIa complex, thereby inhibiting platelet aggregation [9,10]. Thus, clopidogrel is effectively used to reduce secondary vascular events such as thromboembolism in patients with atherosclerosis. Consequently, the combination therapy of rosuvastatin and clopidogrel should be beneficial for more effective management of cardiovascular diseases [11]. In addition to the synergistic effect of the therapeutic outcome, a combination product generally improves patient compliance since it is more convenient to self-administer fewer pills [12,13]. However, developing combination products with rosuvastatin and clopidogrel is not straightforward due to significant instability issues in direct mixing. Since clopidogrel is generally used as a bisulfate salt due to its low solubility and stability [14,15,16], it is strongly acidic, while rosuvastatin is acid-labile [7,8,17]. Therefore, in the present study, a multilayer-coated tablet of clopidogrel and rosuvastatin was prepared to circumvent instability issues caused by mixing rosuvastatin and clopidogrel acidic salt (Figure 2). Its in vitro and in vivo characteristics were then evaluated in comparison with commercially available products.

## 2. Materials and Methods

### 2.1. Materials

Clopidogrel bisulfate and rosuvastatin calcium were purchased from Dr. Reddy’s Laboratory Ltd. (Hyderabad, India) and TEVA Pharmaceutical Industries Ltd. (Petah Tikva, Israel), respectively. Low-substituted hydroxypropyl cellulose (L-HPC) was obtained from Shin-Etsu Chemical (Tokyo, Japan). Copovidone, crospovidone, and sodium lauryl sulfate were purchased from BASF (Ludwigshafen, Germany). Lactose monohydrate (Meggle Pharma, Wasserburg, Germany), colloidal silicon dioxide (Evonik, Essen, Germany), talc (Nippon Talc, Osaka, Japan), magnesium stearate (Mallinckrodt Pharmaceuticals, Staines, UK), carnauba wax (SS Pharm Co., Ansan, South Korea), sodium stearyl fumarate (JRS Pharma, Rosenberg, Germany), glyceryl behenate (Gattefosse, Saint-Priest, France), titanium dioxide (Kronos Inc., Chelmsford, MA, USA), sodium starch glycolate (DFE Pharma, Goch, Germany), and croscarmellose sodium (FMC Co., Philadelphia, PA, USA) were also purchased from the corresponding companies as indicated in parentheses. All other chemicals and reagents were of analytical grade.

### 2.2. Methods

#### 2.2.1. Preparation of Clopidogrel Core Tablets

Clopidogrel core tablets were prepared by direct compression. The composition of each core tablet (F1–F5) is summarized in Table 1. To select the appropriate disintegrant, each core tablet was prepared with and without a super-disintegrant such as sodium starch glycolate, croscarmellose sodium, or crospovidone. All excipients were thoroughly mixed with clopidogrel bisulfate in the predetermined ratios given in Table 1, and then clopidogrel core tablets were prepared via direct compression of these powder mixtures using a concave round punch (8.5 mm diameter) in a tablet press (XENA-I Premium, LAON, Siheung, South Korea).

A disintegration test (described in Section 2.2.4.) was carried out to select the optimal disintegrant.

#### 2.2.2. Screening Coating Materials for Separating Clopidogrel and Rosuvastatin

To select the optimal coating agent that can effectively prevent physicochemical interactions between clopidogrel and rosuvastatin, the clopidogrel core tablet was coated with four different materials (hypromellose, carnauba wax, sodium stearyl fumarate, and glyceryl behenate) following the process described in Section 2.2.3. Then, clopidogrel core tablets coated with each coating material were placed in high-density polyethylene (HDPE) bottles containing rosuvastatin calcium (active pharmaceutical ingredient (API) powder) for four months in accelerated stability test conditions at 40 °C and a relative humidity (RH) of 75%. The effect of hydrophobic coating materials on the appearance of the rosuvastatin impurity, lactone, was determined by HPLC.

#### 2.2.3. Preparation of the Multilayer-Coated Tablet

Preparation of Coating Solutions: The coating solution for the hydrophobic separation layer and the outer layer of rosuvastatin were prepared with the composition described in Table 2. To prepare the coating solution of the hydrophobic separation layer, copovidone was dissolved in a mixture of water and ethanol (75:300, *v*/*v*). The other excipients including glyceryl behenate, titanium dioxide, and talc were well-dispersed in this mixture. Prior to the coating process, this solution was passed through a 70-mesh sieve and stirred continuously during the coating process.

The rosuvastatin coating solution was also prepared using a similar process. All excipients except rosuvastatin calcium and talc were dissolved in a mixture of water and ethanol (70: 200, *v*/*v*) at 40 °C. Then, rosuvastatin calcium was added to this mixture. After the complete dissolution of rosuvastatin, talc was dispersed in the mixed solution. Prior to the coating process, the coating solution was passed through a 70-mesh sieve and stirred continuously at 40 °C during the coating process.

Tablet Coating Process: The clopidogrel core tablet was first coated with the hydrophobic separation layer, and then the rosuvastatin coating solution was overlaid on the hydrophobic separation layer. Each coating process was performed in a similar manner using an HCT-30 Hi-coater (Freund-Vector, Marion, IA, USA). First, tablets were preheated in a coating pan at approximately 45 °C. During the coating process, the coating pan was rotated at a speed of 10–15 rpm, and hot air was supplied to maintain an outlet temperature of 38–44 °C. The coating solution was sprayed at a rate of 5.0–5.5 g/min using a nozzle with a diameter of 1.4 mm and an air pressure of 1.0–1.4 kgf/cm^2^. When the coating process was finished, the coated tablets were dried for 1 h in the coating pan.

#### 2.2.4. Disintegration Study

Disintegration studies were conducted by using a disintegration tester (DIT-200, LABFINE, Anyang, South Korea) as described in the United States Pharmacopeia (USP). One tablet was placed into each tube in the disintegration tester, and the tester was operated in water at 37 ± 2 °C. When all test tablets were completely disintegrated, the test was stopped, and the time required for complete disintegration was recorded.

#### 2.2.5. Dissolution study

Dissolution studies were performed in a dissolution tester DST-810 (LABFINE, Anyang, South Korea) using the USP paddle method at 37 ± 0.5 °C and 50 rpm. Each formulation was exposed to the aqueous dissolution medium at different pHs (1.2, 4.0, and 6.8) for 120 min. At predetermined time points (5, 10, 15, 30, 45, 60, 90, and 120 min), 5 mL of each sample were withdrawn and an equal volume of fresh medium was added into the vessel to maintain a constant volume of dissolution media. The collected samples were filtered through 0.45-µm pore-sized syringe filters. The filtrates were diluted with the mobile phase, and the released drug amount was analyzed by HPLC.

#### 2.2.6. Stability study

Each formulation (reference and test) was stored under accelerated (40 °C/RH 75%) and long-term (25 °C/RH 60%) stability test conditions. After three months in a stability chamber (VP1300, Vötsch Industrietechnik, Balingen, Germany), the production of rosuvastatin impurities (the amount of lactone and total impurities) were determined by HPLC.

#### 2.2.7. Pharmacokinetic Study in Dogs

Animal studies were carried out in accordance with the “Guiding Principles in the Use of Animals in Toxicology” adopted by the Society of Toxicology (USA), and the study protocol was approved by the review committee of Research Institute, Dong Wha Pharm. (IACUC-16028). Prior to the experiments, beagle dogs (10.2–12.2 kg) were fasted for 8 h and divided into two groups (*n* = 4 per group): Group 1 received the reference product, and Group 2 received the test formulation. Plavix^®^ (75 mg) and Crestor^®^ (10 mg) were used as the reference product for clopidogrel and rosuvastatin, respectively. The pharmacokinetic profiles of the reference products and the test formulation (F5b) in dogs were evaluated in a randomized, single dose, 2 × 2 crossover study with a 2-week washout period. Prior to drug administration, the animals were intramuscularly administered pentagastrin to regulate the intragastric pH. After the dogs were orally administered the reference product (two tablets of Plavix^®^ (75 mg) and two tablets of Crestor^®^ (10 mg)) or the test formulation (two tablets of F5b containing clopidogrel (75 mg) and rosuvastatin (10 mg)), blood samples were collected at 0.5, 1, 1.5, 2, 3, 5, and 7 h. Blood samples were centrifuged at 12,000 rpm for 10 min, and the obtained plasma samples were kept frozen at −80 °C until analyzed by liquid chromatography-tandem mass spectrometry (LC-MS/MS).

#### 2.2.8. HPLC Analysis

Clopidogrel: The drug concentration was determined by HPLC (Alliance HPLC system e2695, Waters, Milford, MA, USA) using an Ultron ES-OVM 5 µm column (150 mm × 4.6 mm). The mobile phase consisted of acetonitrile, 0.01M phosphate buffer, and water in a volume ratio of 175:525:300, and the flow rate was 1.0 mL/min. The injection volume was 5 μL, and the UV wavelength was set at 220 nm.

Rosuvastatin: The drug concentration was analyzed by HPLC (Alliance HPLC system e2695, Waters, Milford, MA, USA) using a Nova-Pak 4-µm C18 column (150 mm × 3.9 mm). The mobile phase consisted of acetonitrile, 1% trifluoroacetate, and water in a volume ratio of 37:1:62, and the flow rate was 0.75 mL/min. The injection volume was 10 μL, and the UV wavelength was set at 242 nm.

Rosuvastatin impurities including lactone: Impurities of rosuvastatin including lactone were assessed by using the HPLC assay described above for the quantification of rosuvastatin. Then, the percentage of each impurity was calculated by using the equation below.

% of each impurity = (*r*_u_/*r*_s_) × (*C*_s_ /*C*_u_) × [*M* × (*M*_r1_/*M*_r2_)] × (1/*F*) × 100 (1)

where *r*_u_ = peak response of each impurity from the sample solution, *r*_s_ = peak response of rosuvastatin from the standard solution, *C*_s_ = concentration of rosuvastatin in the standard solution (mg/mL), *C*_u_ = nominal concentration of rosuvastatin in the sample solution (mg/mL), *M* = number of moles of rosuvastatin per mole of rosuvastatin calcium (2), *M*_r1_ = molecular weight of rosuvastatin (481.54), *M*_r2_ = molecular weight of rosuvastatin calcium (1001.14), *F* = relative response factor (see Table 2 in the USP/NF Rosuvastatin Tablets monograph).

#### 2.2.9. LC-MS/MS Analysis

Plasma drug concentrations were determined by LC-MS/MS. Chromatographic separation was performed with a ZORBAX Eclipse plus C18 column (4.6 mm × 50 mm, 3.5 μm, Agilent). For the mobile phase, isocratic elution was conducted with 0.1% formic acid and acetonitrile containing 0.1% formic acid (30:70, *v*/*v*) at a flow rate of 0.8 mL/min. Mass spectrometric detection was performed with an Agilent 6410 Triple Quad mass spectrometer (Agilent Technologies, Santa Clara, CA, USA) using an electrospray ionization source. Rosuvastatin, clopidogrel, and an internal standard (ketoprofen) were detected with m/z 482.1 → 258.1, 322.0 → 212.0, and 255.1 → 209.1, respectively. Calibration curves for rosuvastatin and clopidogrel were constructed using concentrations of 10–400 ng/mL and 10–600 ng/mL, respectively, which displayed good linearity with *r*^2^ values greater than 0.99.

#### 2.2.10. Pharmacokinetic Analysis and Statistical Analysis

Noncompartmental analysis was performed to assess pharmacokinetic parameters. The maximum plasma concentration (*C*_max_) and the time to reach the maximum plasma concentration (*T*_max_) were observed values from the plasma concentration-time profiles. The area under the plasma concentration-time curve (AUC) was calculated by the linear trapezoidal method.

All data are expressed as mean ± standard deviation (SD). Statistical analysis was performed using Student’s *t*-test. A *p*-value < 0.05 was considered statistically significant.

## 3. Results

### 3.1. Screening of Optimal Excipients

Disintegrants: Five different clopidogrel core tablets (F1–F5) were prepared by direct compression with and without the addition of super-disintegrants. The composition of each tablet is presented in Table 1. The disintegration time of each tablet was determined in water to select the optimal disintegrant. As shown in Table 3, the addition of sodium starch glycolate, croscarmellose sodium, and crospovidone in equal amounts (5 mg) reduced the disintegration times to 9.1 min, 8.48 min, and 7.93 min, respectively, while the core tablet (F1) without any super-disintegrant disintegrated in 12.45 min. Furthermore, increasing the amount of crospovidone from 5 to 12 mg achieved a much faster disintegration time of 3.38 min. Therefore, 12 mg of crospovidone was added as a disintegrant into the final composition of the clopidogrel core tablet.

**Figure 3:**
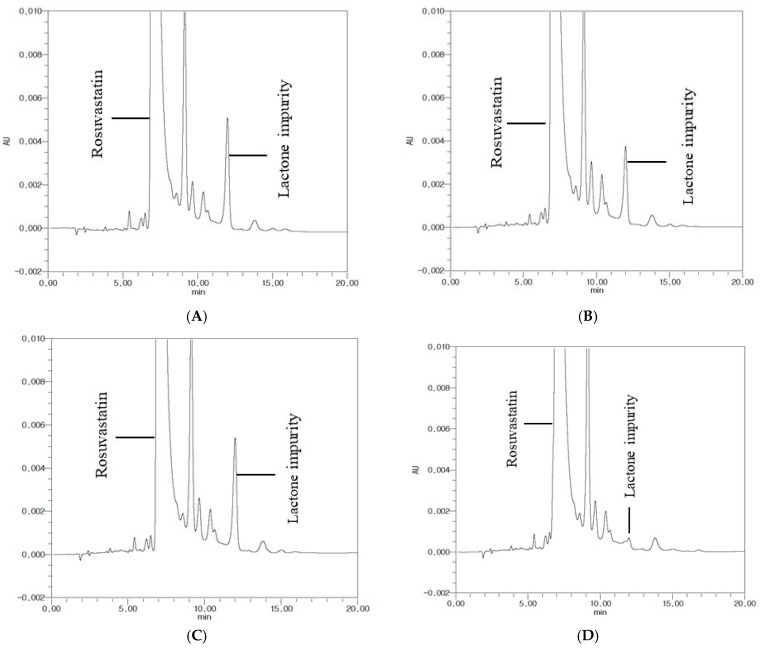
Chromatograms obtained from different formulations after four months of storage at 40 °C/RH 75%. (**A**) Hypromellose, (**B**) carnauba wax, (**C**) sodium stearyl fumarate, and (**D**) glyceryl behenate.

Coating Materials for Clopidogrel Core Tablets: To select the optimal coating material that effectively inhibits the production of lactone, the acid degradation product of rosuvastatin, the clopidogrel core tablet was coated with four different coating materials including hypromellose, carnauba wax, sodium stearyl fumarate, and glyceryl behenate. The resulting coated tablets were placed in high-density polyethylene (HDPE) bottles for four months with rosuvastatin calcium (API powder) under 40 °C and RH 75%. Among the tested materials, hydrophobic glyceryl behenate was the most effective at inhibiting lactone production, which was less than 0.02%. Other tested materials displayed lactone production of 0.166–0.249% (Figure 3). Therefore, glyceryl behenate was selected as the optimal coating material for separating clopidogrel and rosuvastatin.

### 3.2. Dissolution Studies

**Figure 4:**
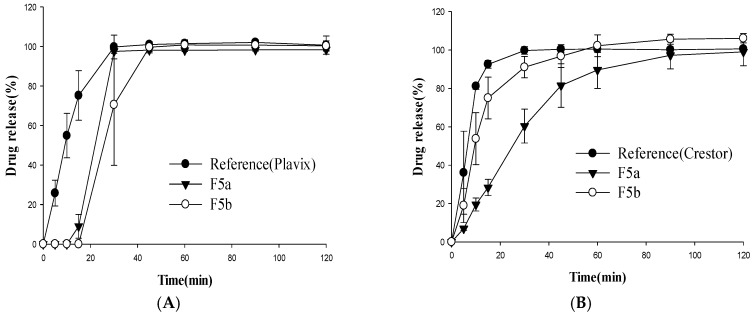
Dissolution profiles of clopidogrel (**A**) and rosuvastatin (**B**) from different formulations at pH 1.2 (mean ± SD, *n* = 4).

The dissolution profiles of the multilayer-coated tablets were examined at different pHs and compared to those from the reference drugs (Plavix^®^ and Crestor^®^). Among the tested tablets, F5b achieved a rapid and almost complete release of rosuvastatin within 1 h at pH 1.2, which was comparable to that of Crestor^®^ (Figure 4). In addition, F5b exhibited a clopidogrel dissolution profile similar to that of Plavix^®^ with the exception of a slight delay at the initial stage of drug dissolution (Figure 4).

**Figure 5:**
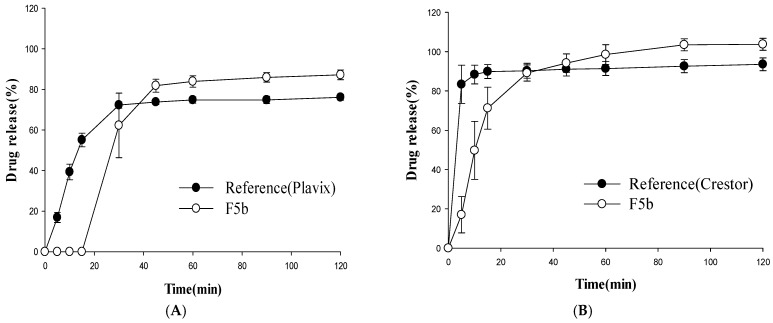
Dissolution profiles of clopidogrel (**A**) and rosuvastatin (**B**) from the test (F5b) and reference formulations at pH 4.0 (mean ± SD, *n* = 4).

**Figure 6:**
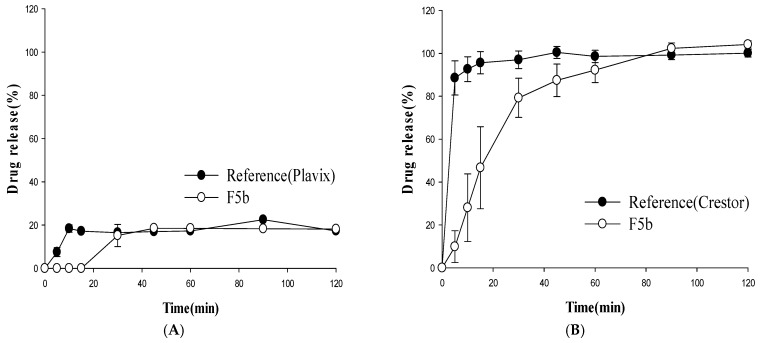
Dissolution profiles of clopidogrel (**A)** and rosuvastatin (**B**) from the test (F5b) and reference formulations at pH 6.8 (mean ± SD, *n* = 4).

**Figure 7:**
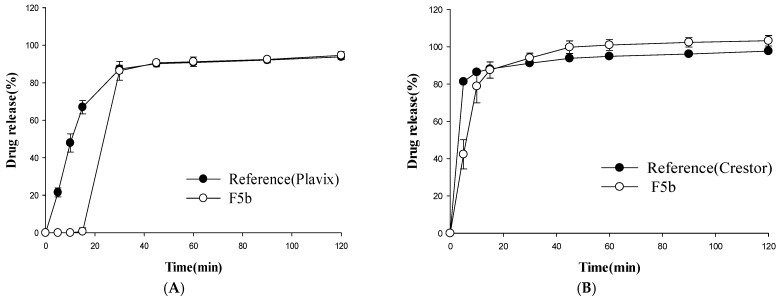
Dissolution profiles of clopidogrel (**A**) and rosuvastatin (**B**) from the test (F5b) and reference formulations in water (mean ± SD, *n* = 4).

The drug release profiles of F5b were also examined at other pHs. Similar to the observation at pH 1.2, F5b displayed clopidogrel dissolution profiles comparable to that of the reference drug for all tested dissolution media (Figure 5, Figure 6 and Figure 7). However, clopidogrel release from F5b was delayed by 15 min at the initial dissolution stage. In the case of rosuvastatin, F5b exhibited rapid and almost complete drug release within 2 h at all tested pHs, although its initial drug release rates were somewhat slower than those of Crestor^®^ (Figure 5, Figure 6 and Figure 7). Overall, the results indicate that F5b dissolution profiles with both APIs (clopidogrel and rosuvastatin) are comparable to those of the reference drugs.

### 3.3. In Vitro Stability Study

The in vitro stability of rosuvastatin in different formulations (F5b and Crestor^®^) was examined under accelerated (40 °C/RH 75%) and long-term (25 °C/RH 60%) stability test conditions. As summarized in Table 4, after three months of storage at 40 °C and RH 75%, the amount of lactone (the acid degradation product of rosuvastatin) was 0.28% and 0.76% for F5b and Crestor^®^, respectively, while the total impurity amounts were 0.78% for F5b and 1.21% for Crestor^®^. Likewise, at 25 °C and RH 60%, the F5b retained the rosuvastatin stability similar to that of the reference product in terms of the amounts of total impurities and lactone. Particularly, the increase in lactone and total impurities during the storage was minimal with F5b at both storage conditions. Collectively, F5b could retain the good stability of rosuvastatin by effectively separating clopidogrel from rosuvastatin via a hydrophobic coating layer.

### 3.4. Pharmacokinetic Studies in Dogs

**Figure 8:**
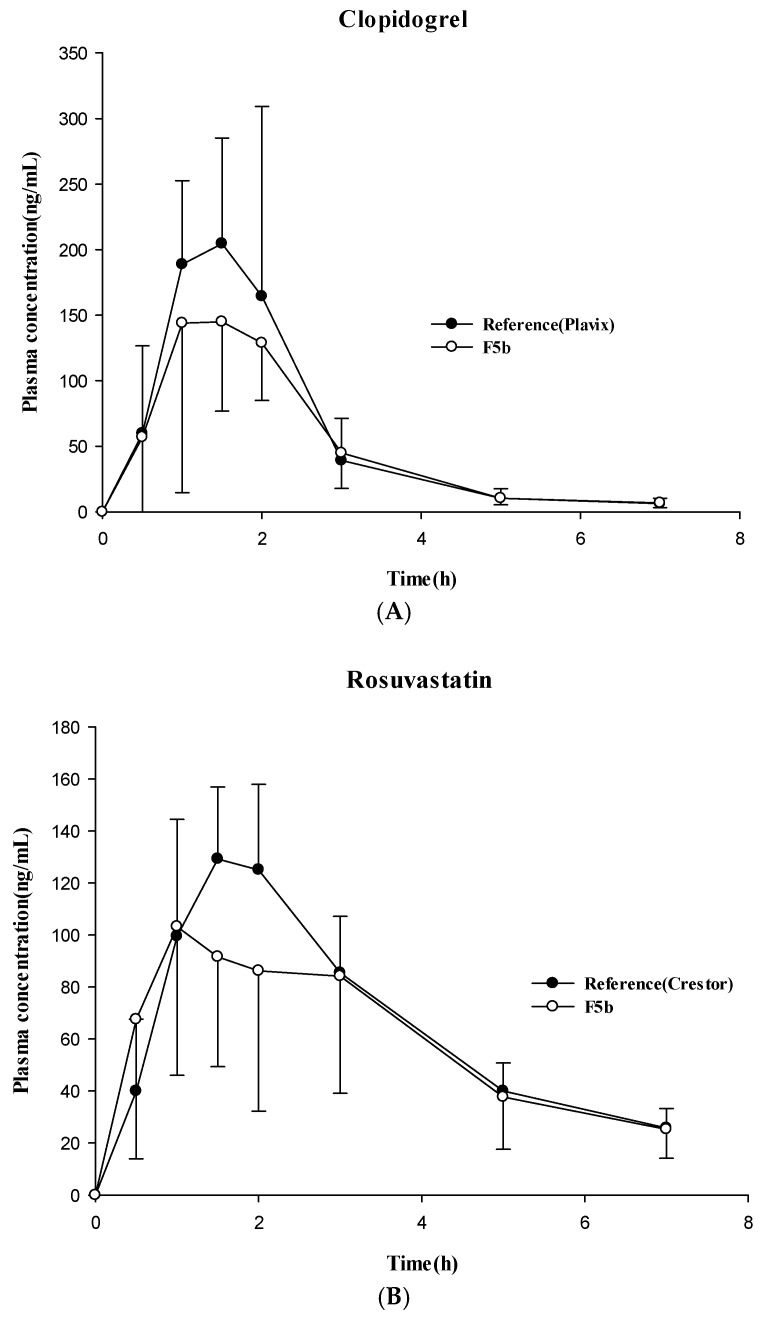
Plasma concentration-time profiles of clopidogrel (150 mg) (**A**) and rosuvastatin (20 mg) (**B**) after oral administration of two different formulations (reference and test) in dogs (mean ± SD, *n* = 8). Reference: Plavix^®^ (75 mg) two tablets and Crestor^®^ (10 mg) two tablets, Test (F5b): two tablets (each containing clopidogrel 75 mg and rosuvastatin 10 mg).

After oral administration to dogs, the pharmacokinetic profiles of F5b were evaluated and compared to those from the reference drugs (Plavix^®^, Crestor^®^). The pharmacokinetic parameters and plasma-concentration profiles are summarized in Table 5 and Figure 8. In the case of clopidogrel, F5b exhibited a systemic drug exposure equivalent to that of Plavix^®^ (Figure 8A), and there was no significant difference between the test and the reference drugs regarding their pharmacokinetic parameters such as *C*_max_, *T*_max_ and *AUC* (Table 5). Similarly, F5b also exhibited the oral exposure of rosuvastatin comparable to that of the reference product, Crestor^®^ (Table 5, Figure 8B), displaying no statistical differences between the test and the reference groups.

Overall, after oral administration in dogs, F5b achieved the systemic exposures of clopidogrel and rosuvastatin equivalent to those of the corresponding reference drugs.

## 4. Discussion

In this study, a multilayer-coated tablet combining clopidogrel and rosuvastatin was developed to inhibit HMG-CoA reductase and thrombosis. Since rosuvastatin is acid-labile, a simple mixed tablet or a double-layered tablet comprised of rosuvastatin and acidic clopidogrel bisulfate is not feasible. The preliminary study on a double-layered tablet without the coating layer also indicated the destabilization of rosuvastatin with direct contact with clopidogrel, leading to the significant production of lactone (data not shown). Therefore, as illustrated in Figure 2, to prevent the acidic destabilization of rosuvastatin in the presence of clopidogrel bisulfate, a hydrophobic coating layer was incorporated into the multilayer coated tablet for the physicochemical separation of the clopidogrel core tablet from an outer rosuvastatin layer. Considering the therapeutic dose levels, clopidogrel (75 mg), which has a relatively high therapeutic dose, was prepared as a core tablet, while rosuvastatin (10 mg) was placed into the outer coating layer.

Since the effectiveness of the clopidogrel core tablet surface coating layer is critical in preventing acid destabilization of rosuvastatin, the clopidogrel core tablet was coated with four different coating materials. The resulting tablets were stored in rosuvastatin powder at 40 °C and RH 75% for four months, and the appearance of the acidic degradation product, lactone, was monitored by HPLC. As shown in Figure 3, among the tested coating materials, glyceryl behenate inhibited the production of lactone most effectively, and the amount of lactone produced was negligible during storage under the accelerated stability test condition. Glyceryl behenate is a lipid excipient widely used for lubrication, taste-masking, and sustained drug release [18,19]. When a lipid-based material such as glyceryl behenate is used as a coating agent, a granule or matrix mixture containing API is prepared by a hot-melt method in most cases. In those cases, drug release is generally slow because of the diffusion process through the lipid matrix [20,21]. Therefore, the coating process in this study was performed with a basic tablet coating method rather than a hot-melt method to minimize the delay of drug release. In addition, the super-disintegrant, crospovidone, was incorporated into the core tablet to prevent the delay of drug release and achieved the rapid disintegration within 3–4 min (Table 3). The major disintegration mechanism of crospovidone is not clearly defined; however, it has been proposed that a complex mechanism including wicking, high swelling pressure, and strain recovery is involved in the rapid disintegration by crospovidone [22,23]. As a result, the clopidogrel core tablet also exhibited rapid drug dissolution in various dissolution media. Its dissolution was comparable to Plavix^®^ with the exception of a 30 min lag time at the initial dissolution stage (Figure 5, Figure 6 and Figure 7).

Regarding the outer coating layer containing rosuvastatin, the addition of solubilizers was necessary to achieve rapid dissolution similar to Crestor^®^. Most solubilizers are liquid or semi-solid at the high temperatures of the tablet coating process. Therefore, copovidone was an ideal solubilizer for the outer coating layer since it has a high melting point of 150 °C and remained in a solid form during the tablet coating process [24,25,26]. In addition, copovidone is effective at increasing drug dissolution [26,27]. Consequently, the dissolution of the rosuvastatin-containing outer coating layer was enhanced as the amount of copovidone was increased (Figure 5). F5b instigated the complete dissolution of rosuvastatin within 2 h in various dissolution media, although its initial dissolution was slightly slower than that of Crestor^®^ (Figure 5, Figure 6 and Figure 7).

F5b was also successful at retaining the rosuvastatin stability in the presence of clopidogrel bisulfate under accelerated (40 °C/RH 75%) and long-term (25 °C/RH 60%) stability test conditions (Table 4). Therefore, F5b was selected as an optimal multi-layered coated tablet, and its pharmacokinetic profiles were evaluated in dogs along with the reference commercial products (Plavix^®^ and Crestor^®^) for comparison. As summarized in Table 5 and Figure 8, F5b achieved the systemic exposure equivalent to the reference products without any statistical difference in *C*_max_, *T*_max_, or *AUC* between the treatment groups. These results suggest that F5b, a multi-layer coated tablet, might be an effective alternative for the combination therapy of Plavix^®^ and Crestor^®^.

## 5. Conclusions

In this study, the multilayer-coated tablet (F5b) was successfully developed as a stable and effective combination product of clopidogrel and rosuvastatin. The storage stability tests showed that F5b could retain the good stability of rosuvastatin by effectively separating clopidogrel from rosuvastatin via a hydrophobic coating layer. Furthermore, F5b achieved the systemic exposure equivalent to the reference products (Plavix^®^ and Crestor^®^) in beagle dogs. Taken together, F5b appeared to be a stable multilayer-coated tablet of clopidogrel and rosuvastatin that may improve the patient compliance in combination therapy for cardiovascular diseases.

## Figures and Tables

**Table 1:** Composition of the clopidogrel bisulfate core tablet.

Materials (mg)	F1	F2	F3	F4	F5
Sodium starch glycolate		5.0			
Croscarmellose sodium			5.0		
Crospovidone				5.0	12
Clopidogrel bisulfate	97.9	97.9	97.9	97.9	97.9
L-HPC	25.0	25.0	25.0	25.0	25.0
Lactose	110.1	105.1	105.1	105.1	105.1
Copovidone	12.0	12.0	12.0	12.0	12.0
Colloidal silicon dioxide	2.5	2.5	2.5	2.5	2.5
Talc	4.0	4.0	4.0	4.0	4.0
Magnesium stearate	3.5	3.5	3.5	3.5	3.5
Total weight	255.0	255.0	255.0	255.0	262.0

**Table 2:** Composition of the coating solution.

Coating Layer	Materials (mg)	F5a	F5b
Hydrophobic separation coating layer	Glyceryl behenate	15	15
	Copovidone	10	10
	Titanium dioxide	0.5	0.5
	Talc	0.5	0.5
	Water	75	75
	Ethanol	300	300
Rosuvastatin coating layer	Rosuvastatin calcium	10.4	10.4
	Copovidone	39.6	59.6
	Talc	2.0	2.0
	Water	70.0	70.0
	Ethanol	200.0	200.0

**Table 3:** Disintegration time of the clopidogrel bisulfate core tablet in water at 37 °C.

Disintegrants	Disintegration Time (min)
Sodium starch glycolate	9.1
Croscarmellose sodium	8.48
Crospovidone	7.93
Crospovidone (12 mg/tablet)	3.38

**Table 4:** Three month-storage stability of the multilayer-coated tablet (F5b) under different conditions (mean ± SD, *n* = 3).

Impurity	Formulation	Rosuvastatin Impurity Amount (%)		
		40 °C /RH 75%		25 °C/RH 60%
		0 Month	3 Months	3 Months
Lactone	Crestor^®^	0.11±0.07	0.76±0.14 *	0.14±0.02
	F5b	0.15±0.01	0.28±0.03 *	0.19±0.01
Total impurity	Crestor^®^	0.43±0.06	1.21±0.14 *	0.66±0.04 *
	F5b	0.73±0.02	0.78±0.04	0.72±0.01

* *p* < 0.05, compared to that at 0 month.

**Table 5:** Pharmacokinetic parameters of clopidogrel (150 mg) and rosuvastatin (20 mg) after an oral administration of two different formulations (reference and test) in dogs (mean ± SD, *n* = 8).

Drug	Parameters	Formulations	
		Reference	Test (F5b)
Clopidogrel	*C*_max_ (ng/mL)	249 ± 90.0	224 ± 107
	*T*_max_ (h)	1.38 ± 0.52	1.44 ± 0.56
	*AUC* (ng·h/mL)	436 ± 197	364 ± 138
Rosuvastatin	*C*_max_ (ng/mL)	139 ± 24.7	122 ± 47.6
	*T*_max_ (h)	1.56 ± 0.32	1.38 ± 0.83
	*AUC* (ng·h/mL)	462 ± 107	423 ± 214

Reference formulation: Plavix^®^ (75 mg) two tablets and Crestor^®^ (10 mg) two tablets; Test (F5b) formulation: Two tablets (each containing clopidogrel 75 mg and rosuvastatin 10 mg).
